# The complete mitochondrial genome of *Hemerobius spodipennis* (Neuroptera: Hemerobiidae)

**DOI:** 10.1080/23802359.2020.1764400

**Published:** 2020-05-18

**Authors:** Yang Zhao, Jinquan Jing, Ningning Zhang, Heping Shao

**Affiliations:** Nanjing Institute of Agricultural Sciences in Jiangsu Hilly Area, Nanjing, P.R. China

**Keywords:** *Hemerobius spodipennis*, mitochondrial genome, Hemerobiidae, phylogenetic relationships

## Abstract

The complete mitochondrial genome of *Hemerobius spodipennis* Yang, 1987 was sequenced in this study. The complete mitochondrial genome is a typical double-stranded circular molecule of 16,343 bp (GenBank accession number: MT268963) comprising of 13 protein-coding genes, 22 transfer RNA genes, 2 ribosomal RNA genes, and a control region. The gene order is identical to that of the putative ancestral arrangement of insects and other lacewings. All protein-coding genes initiate with ATN, except *COI* use CGA as start codons and terminate with TAG or TAA, expect *ND5* and *ND4* use TA– or a single T–– residue as the stop codon. All tRNAs, ranging from 63 to 72 bp, can be folded into typical clover-leaf secondary structure except for *tRNA^Ser(AGN)^*, in which the dihydrouridine (DHU) arm did not form a stable stem-loop structure. The control region is 1433 bp long with an A + T content of 91.4%. In the sampled families of Neuroptera, each family showed a monophyletic cluster and Polystoechotidae + Rapismatidae, Hemerobiidae + (Chrysopidae + (Polystoechotidae + Rapismatidae)), are recovered in phylogenetic analyses with high supports.

Hemerobiidae, the brown lacewings, is the third largest family of Neuroptera, with about 650 species in the world and widely distributed. The genus *Hemerobius* is the largest genus of family Hemerobiidae, with about 250 species in the world (Oswald 1993, 2019; Monserrat 2000). In this study, we present the complete mitochondrial genome of the Chinese specie *Hemerobius spodipennis* Yang, 1987, which belongs to genus *Hemerobius* and important natural enemies because both their adults and larvae prey on aphid, scale insect, worm eggs, and mollusk insects (Yang 1981). The samples were collected in Purang County, Xizang, China (30°12′4″N, 81°15′10″E). Voucher specimen (No. HEME-00813) was deposited at the Entomological Lab of Nanjing Institute of Agricultural Sciences.

This mitochondrial genome is 16,343 bp long (GenBank accession number: MT268963). It includes the entire set of 37 genes (i.e. 13 protein-coding genes, 22 transfer RNA genes, and 2 ribosomal RNA genes) usually present in animal mitochondrial genomes and a control region. Gene order is identical to that of the putative ancestral arrangement of insects and other lacewings (Haruyama et al. 2011; Zhao et al. 2013; Cameron 2014; Zhang and Wang 2016; Zhao et al. 2016, 2020). There are a total of 43 overlapped nucleotides between genes in 16 locations, ranging from 1 to 7 bp in length; while there are totally 1694 bp intergenic nucleotides in 14 locations, ranging from 1 to 1433 bp in length.

ATN, GTG, TTG and GTT are accepted canonical mitochondrial start codons for invertebrate mtDNAs and most of the PCGs exhibit these start codons (Wolstrnholme 1992). Twelve protein-coding genes initiate with ATN as the start codon (ATG for *COII, ATP6, COIII, ND4, ND4L* and *Cytb*, ATT for *ND2, ATP8, ND3, ND5, ND6*, ATA for *ND1*). The exception is *COI* gene, which uses CGA as start codon. Conventional stop codons TAG and TAA are respectively distributed to two and nine protein-coding genes. However, *ND5* terminates with TA–, and *ND4* uses a single T–– residue as the stop codon.

There are 22 tRNA genes, ranging from 63 to 72 bp in length, and all of them can be folded into typical clover-leaf secondary structure expect for *tRNA^Ser(AGN)^*, the dihydrouridine (DHU) arm of which forms a simple loop, as is common phenomenon in most insects. The length of *lrRNA* and *srRNA* is 1,324 bp and 784 bp, respectively. The A + T content of *lrRNA* and *srRNA* are determined to be 84.4% and 82.1%.

The control region is located between srRNA and *tRNA^Ile^* and is 1433 bp in length with an A + T content of 91.4%, which is the most AT-rich region of this mitogenome. The A + T content of the whole genome, PCGs, tRNAs, and rRNAs was 80.2%, 78.2%, 79.3%, and 83.6%, respectively.

Phylogenetic relationship was inferred from phylogenetic analysis of the 13 protein-coding genes and generated by the neighbor-joining method (NJ) of MEGA7.0. Phylogenetic analyses showed the similar relationships among sampled families as shown in Winterton et al. (2010). Each family showed a monophyletic cluster and the following clades were highly supported (Figure 1): (1) Polystoechotidae + Rapismatidae; and (2) Hemerobiidae+(Chrysopidae+(Polystoechotidae + Rapismatidae)).

**Figure 1.**
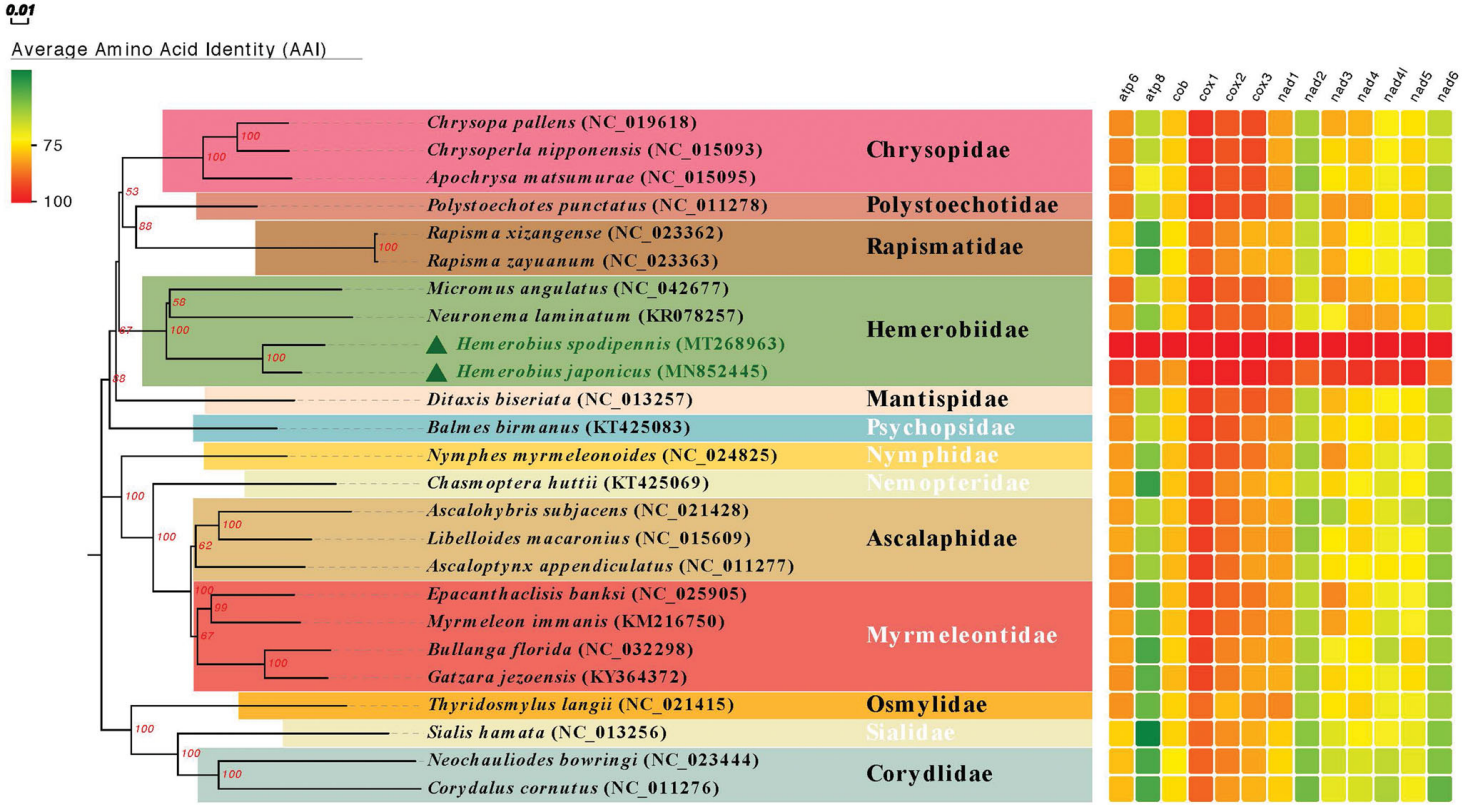
Phylogenetic relationship of seven Neuroptera families which was inferred from phylogenetic analysis of the 13 protein-coding genes and generated by neighbor-joining method (NJ) of MEGA 7.0. Number above each node indicates the bootstrap support values with 1000 replicates.

## Data Availability

The data that support the findings of this study are openly available in NCBI at https://www.ncbi.nlm.nih.gov/, reference number MT268963.
